# Acute Limb Ischemia in a COVID-19 Patient: A Case Report

**DOI:** 10.3400/avd.cr.21-00090

**Published:** 2021-12-25

**Authors:** Naohiro Akita, Takuya Osawa, Hirona Todoroki, Keisuke Mizuno

**Affiliations:** 1 Department of Vascular Surgery, Toyota Kosei Hospital, Toyota, Aichi, Japan

**Keywords:** COVID-19, acute limb ischemia, arterial thrombosis

## Abstract

A 63-year-old man with a confirmed case of coronavirus disease 2019 and having complaints of severe pain and paralysis in his right lower limb was transported to our hospital in an ambulance. Because of thrombosis, a computed tomography angiogram revealed the occlusion of right common iliac artery and stenosis of abdominal aorta. Emergency angiography and thrombectomy were performed; after surgery, the patient was managed in the intensive care unit with mechanical ventilation and hemodialysis for renal failure. However, on postoperative day 7, thrombosis recurred, and he died because of multiple organ failure.

## Introduction

Coronavirus disease 2019 (COVID-19) is caused by the novel severe acute respiratory syndrome coronavirus 2.^1)^ After the COVID-19 outbreak in Wuhan, China, >170 million infections and 3.6 million deaths have been reported worldwide from December 2019 to June 1, 2021.^2)^

The primary symptoms of this disease are cold symptoms, fever above 37.5°C for >4 days, severe fatigue, acute distress, and respiratory failure; however, certain studies demonstrated that patients with COVID-19 have hypercoagulability, which is an emerging complication that might be associated with high mortality.^3)^ Commonly, venous thromboembolism (VTE) is reported in patients hospitalized with severe COVID-19; however, arterial thromboembolism has been reported as a rare form of thrombosis.^4–6)^ In this study, we report a case of acute limb ischemia (ALI) because of arterial thrombosis associated with COVID-19.

## Case Report

**Figure 1:**
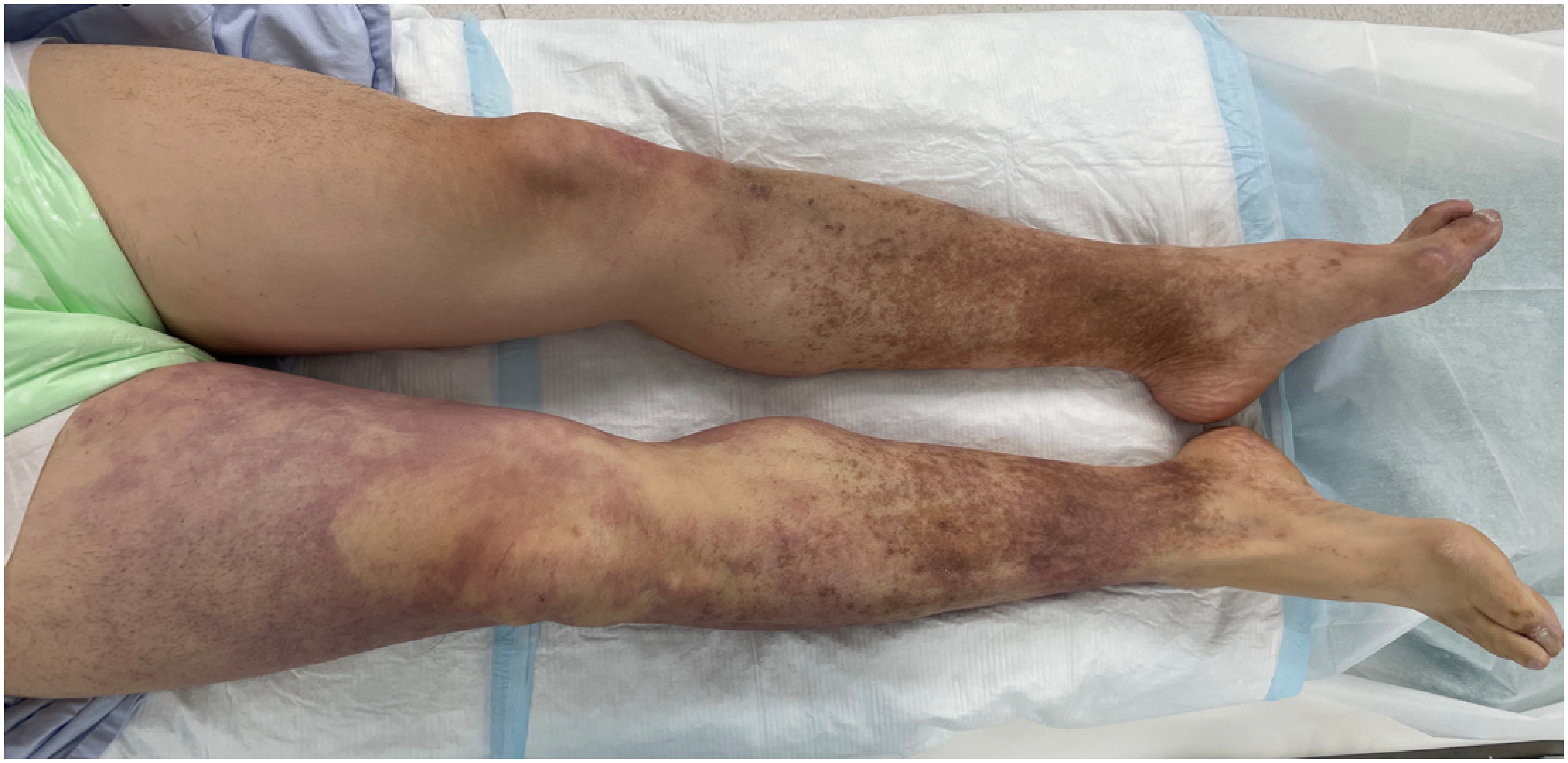
Fig. 1 Right leg had prominent coldness and pallor at the time of presentation.

A 63-year-old man went to a general practitioner with the complaints of some fever and sore throat. His past medical history was not relevant; there was no previous medical history of atrial fibrillation, VTE, atherosclerosis obliterans, cardiovascular disease, or cerebrovascular disease. Furthermore, there was no family history of thrombophilia, and blood tests demonstrated no thrombophilia in this patient. The patient underwent an antigen quantification test, which was positive for COVID-19; he did not have severe symptoms and stayed home and isolated himself from others. After twelve days, he was transported to our hospital in an ambulance because of severe pain and paralysis in his right lower limb. The patient’s right leg demonstrated prominent coldness, pallor, and purpura, with motor and sensory deficits in the lower leg (**Fig. 1**). There were no abnormal neurological results in the thigh; there was no muscle rigidity in the right lower extremity. The right femoral, popliteal, and pedal pulses were absent. There was no Doppler signal in either the right dorsalis pedis or the posterior tibial artery. In the left leg, femoral, popliteal, and pedal pulses were detectable. Physical examination demonstrated a temperature of 36.0°C, blood pressure of 119/94 mmHg, and heart rate of 143 beats per minute. His respiratory rate was 35 breaths per minute, and his oxygen saturation was 92% with 10 l/min of supplemental oxygen; however, he was not in acute distress. On examination, there were no results suggestive of VTE. The polymerase chain reaction (PCR) test for COVID-19 confirmed an active infection.

During initial laboratory evaluations, the following values were noted: white blood cells, 15,800 (normal: 3,300–8,600); creatinine, 0.97 mg/dl (reference: 0.65–1.07 mg/dl); blood urea nitrogen, 48 mg/dl (reference: 8–20 mg/dl); D-dimer, 70.1 (reference: <0.5); antithrombin III, 88.1% (reference: 75%–125%); and C-reactive protein, 9.62 mg/dl (reference: <0.14 mg/dl).

**Figure 2:**
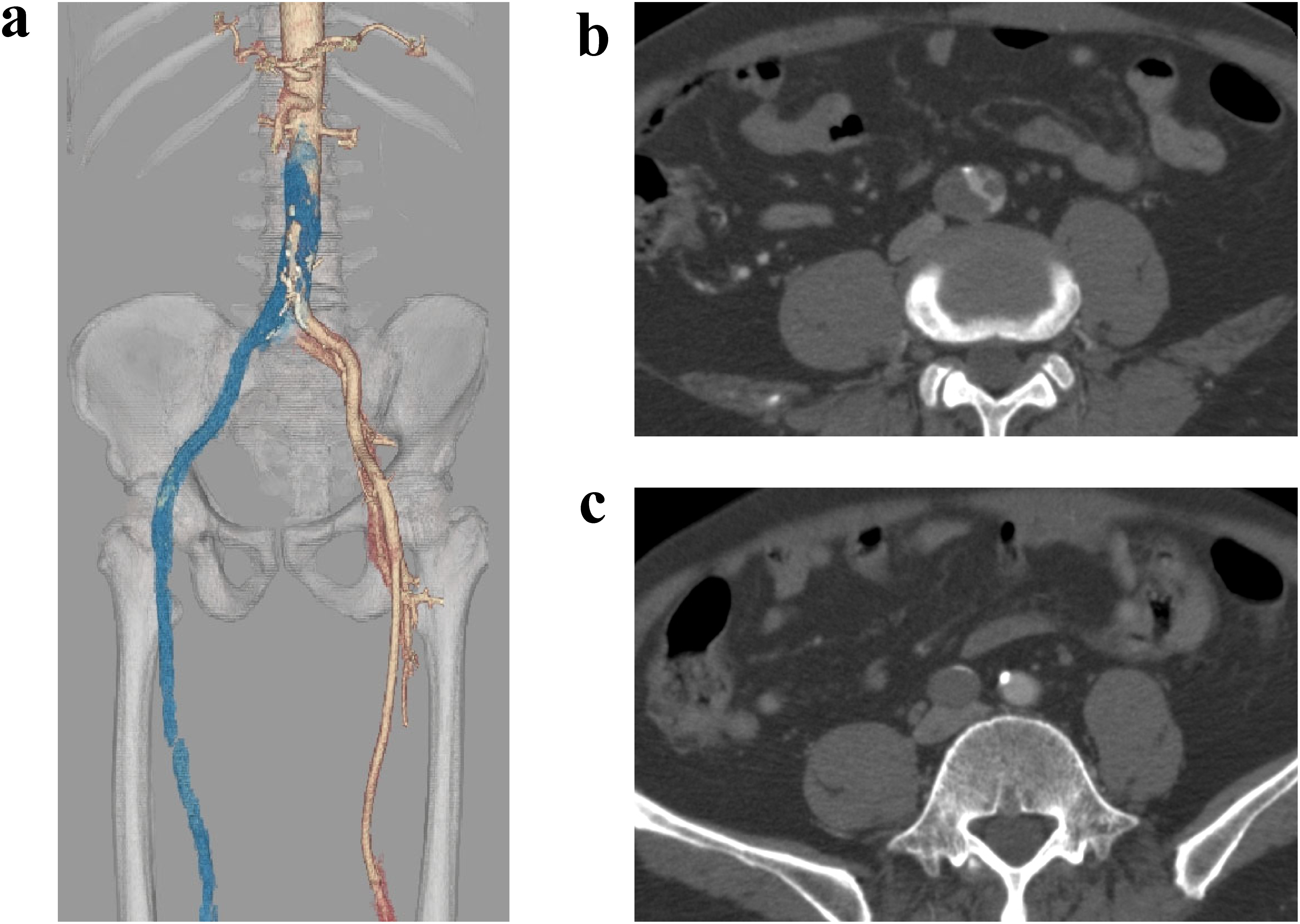
Fig. 2 Three-demensional reconstruction of computed tomography angiography demonstrated occlusion of the right common iliac artery, which was colored light blue (**a**). There was a stenosis because of thrombosis in the infrarenal abdominal aorta (**b**). The right common iliac artery with no contrast in the vessels distal to the occlusion (**c**).

He underwent computed tomography (CT) angiography; the CT angiogram demonstrated acute thrombosis of right common iliac artery with no contrast in the vessels distal to the occlusion (**Fig. 2**). Moreover, there was a stenosis because of thrombosis in the infrarenal abdominal aorta. Chest X-ray demonstrated infiltrates in both lungs. A chest CT scan demonstrated bilateral multiple ground-glass opacities, which is typical of COVID-19 pneumonia.

After there were no collateral blood vessels, we made a diagnosis of ALI (Rutherford III) and severe COVID-19 pneumonia. Therefore, emergency angiography and thrombectomy were planned. Immediately, 5000 IU of unfractionated heparin (UFH) was intravenously administered, and he was transferred to the operating room for surgery.

Because the patient’s distress worsened when he was transported, the surgery started after inducing general anesthesia.

**Figure 3:**
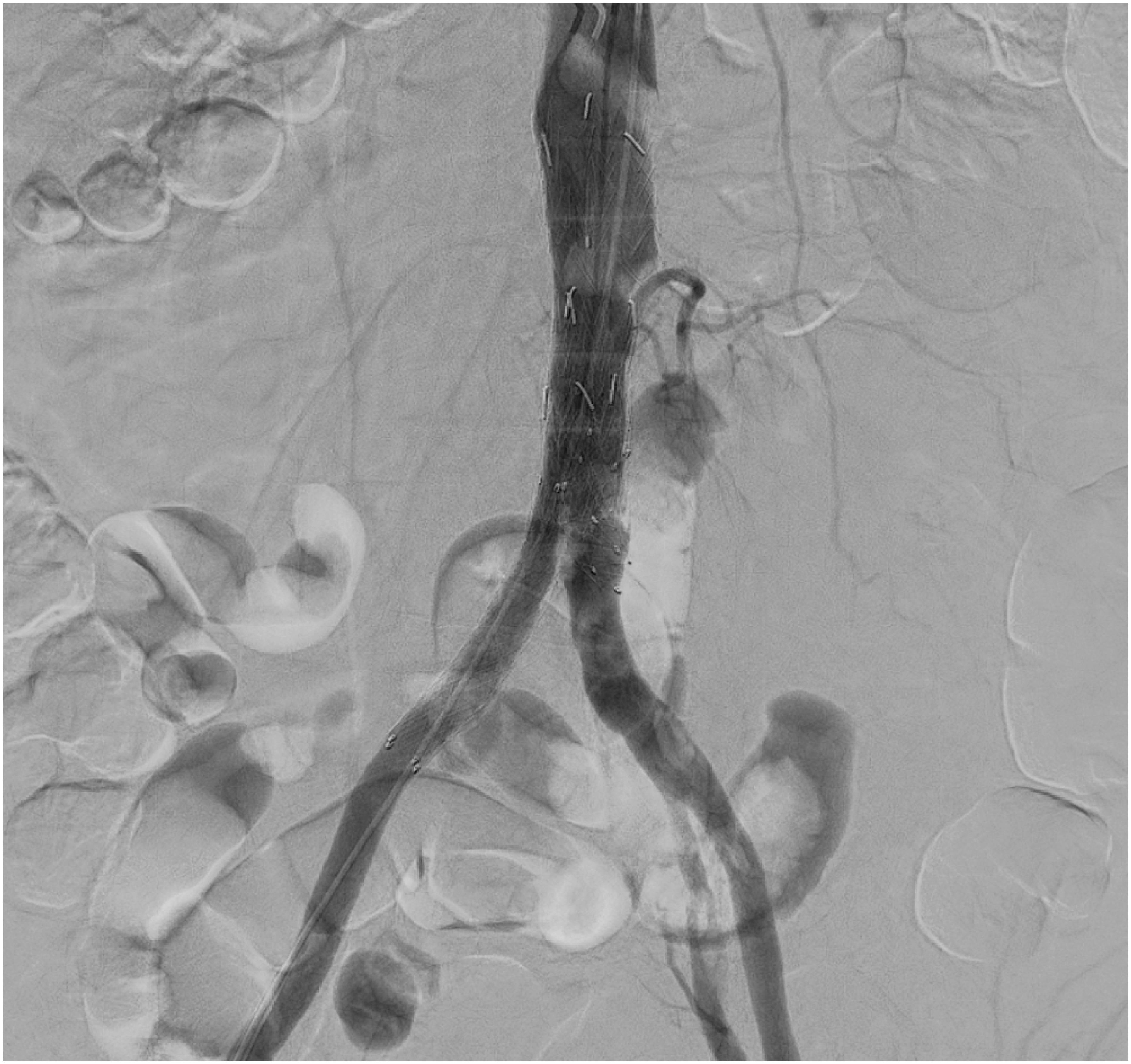
Fig. 3 Intraoperative angiography after embolectomy and stent replacement revealed blood flow improvement in the right lower extremity.

We performed thrombectomy via the bilateral femoral arteries. After the clot was removed from the aorta, bilateral iliac arteries, and right superficial femoral artery using 4 Fr. Fogarty catheters and back bleeding was improved. The diagnostic angiogram demonstrated a patent from the right common iliac artery to the popliteal artery; however, thrombus was present in the abdominal aorta and proximal bilateral iliac arteries. To prevent aorto-iliac occlusion and distal embolism, aortic cuffs (Excluder; W. L. Gore & Associates, Flagstaff, AZ, USA) and self-expanding stents were placed in the abdominal aorta and bilateral common iliac arteries (**Fig. 3**). After surgery, the patient was transferred to the intensive care unit, and intravenous UFH infusions were started. On the first postoperative day (POD1), the patient’s respiratory function worsened; continuous high positive airway pressure was required. Hemodialysis was immediately initiated owing to acute renal failure caused by rhabdomyolysis after surgery. The results of blood tests before surgery and during hospitalization are shown in **Table 1**. Moreover, his creatine kinase level was remarkably high and peaked on POD4 with a value of 152448 U/l. On POD3, the circulation status stabilized and catecholamines were no longer administered; however, his renal function did not improve and continuous hemodialysis was required. Moreover, his respiratory status did not improve. On POD7, although the therapeutic doses of UFH were continuously administered, the patient developed coldness and pallor of the left upper limb, and Doppler ultrasonography revealed the occlusion of brachial artery. Despite sustained hemodialysis, acidosis, hyperkinemia, and liver failure rapidly developed. Finally, his blood pressure and respiratory status worsened, and the patient unfortunately died of multiple organ failure.

**Table 1:** Table 1 Laboratory results from hospitalization days 1 to 7

	Day 0	Day 1	Day 3	Day 5	Day 7	Normal range
White blood cell count, 10^3^/µl	15.8	26.8	15.3	25.1	31.0	3.3–8.6
Neutrophil count, 10^3^/µl	13.9	25.7	14.2	23.6	29.5	—
Lymphocyte count, 10^3^/µl	0.9	0.3	0.5	0.8	0.3	—
Platelet count, 10^4^/µl	20.0	15.1	5.6	5.3	8.7	15.8–34.8
Hemoglobin, g/dl	16.2	11.7	12.1	12.2	13.8	13.7–16.8
Prothrombin time, s	15.5	15.3	19.8	15.6	17.3	9.8–12.1
Activated partial thromboplastin time, s	36.5	56.8	44.3	89.3	92.2	25.0–38.0
C-reactive protein, mg/dl	9.62	7.04	16.29	12.96	9.66	<0.14
D-dimer, µg/ml	70.1	45.2	39.2	32.1	25.7	<1.0
Fibrinogen, mg/dl	189	137	286	404	588	170–410
Creatine kinase, IU/l	12,178	53,924	128,743	66,840	44,199	59–248
Serum creatinine, mg/dl	0.97	1.49	2.83	3.88	3.92	0.65–1.07

## Discussion

Our study describes a confirmed case of COVID-19 complicated with ALI. To date, the reports of surgical treatment for arterial thrombosis in COVID-19 are extremely rare in Japan. This helps expand the available evidence that COVID-19 is associated with incident acute arterial thrombosis.

In this case, we diagnosed category 3 ALI, and we decided to proceed with thrombectomy and planned to perform a two-stage lower extremity amputation after the demarcation line was identified. At the time of admission, the extent of ischemia in the right lower extremity was unclear, making it difficult to determine the amputation line. Permanent neuropathy was then suspected in the right lower leg but that in the thigh seemed to be reversible with limited neuropathy in both movement and sensation. With the occlusion of all peripheral vascular beds distal to the right common iliac artery, it was considered that healing might not be possible even with femoral amputation without revascularization. Furthermore, if revascularization successfully preserved as much of the lower limb as possible, a calf amputation was more beneficial for using a prosthesis than a hip dissection or thigh amputation. The myo-renal metabolic syndrome associated with reperfusion could be controlled with the early introduction of dialysis and careful management. Unfortunately, the patient’s general condition did not improve before the second-stage amputation could be performed.

One study reported that patients admitted with COVID-19 developed ALI at a rate of 0.9%,^4)^ which was lower than the VTE rate^7)^ but much higher than that in the general population.^8)^ The patients were innately healthy and did not have common risk factors for ALI such as any thrombotic predisposition, atrial fibrillation, or peripheral vascular disease. This result agrees with previous reports.^4,7)^

In this case, the patient developed ALI 12 days after receiving a COVID-19 diagnosis. Furthermore, Topcu et al. reported the ALI onset in 11–14 days.^7)^ Note that ALI in COVID-19 patients may occur later in hospitalization and be accompanied by worsening respiratory function. In patients with COVID-19, the close monitoring of D-dimer level to screen for hypercoagulability is recommended; if there is an increasing trend, the introduction of heparin should be considered early in the clinical course.

We then performed revascularization and postoperative anticoagulant therapy primarily with UFH; however, on POD7, the patient died because of multi-organ failure.

Bellosta et al. performed 17 revascularization procedures in 20 patients with confirmed COVID-19 cases complicated with ALI; revascularization was successful in 12 cases, but two patients developed recurrent thrombotic occlusion and required reintervention.^6)^ In total, 14 limbs were salvaged. Eight patients died in the hospital, including four patients with acute respiratory distress syndrome (ARDS), two patients with multiple organ failure, one patient with acute respiratory failure, and one patient with acute myocardial infarction. Sánchez et al. reported 30 patients with ALI in Peru^5)^ in a multicenter retrospective study. Note that twenty-three patients underwent surgery for ALI (Rutherford class IIA 23, IIb 5, III 2); of these patients, two required reinterventions. Nine patients required amputation and seven patients died. ARDS was the cause of all deaths. In a retrospective review of 2,943 patients with COVID-19, Gonzalez-Fajardo et al. reported that patients with ALI had a higher risk of death than patients with other thrombotic events such as deep vein thrombosis and pulmonary embolism. Univariate analysis revealed a 10.3-fold increased risk of death after peripheral arterial thrombosis; moreover, multivariate analysis revealed a 7.5-fold increased risk of death after peripheral arterial thrombosis.^9)^ Their study suggested that COVID-19 severity rather than the success of revascularization might be related to mortality. Moreover, arterial thrombosis was associated with higher mortality rates than was VTE. Furthermore, they reported that the recurrence of arterial thrombosis despite continued anticoagulation, as in our case, was catastrophic. Arterial thrombosis was caused by hypercoagulation because of COVID-19, which is extremely difficult to treat.

The pathogenesis by which COVID-19 patients are prone to arterial thrombosis is not yet clear. Patients may develop acute thrombosis in native, non-atherosclerotic arteries. Gonzalez-Fajardo et al. proposed that severe inflammation damages the endothelium of macrovessels and microvessels, which disrupts normal anti-thrombotic and anti-inflammatory mechanisms, thus resulting in the excessive activation of the thrombotic cascade.^9)^ The thrombosis of the left upper limb occurred on the POD7 when the dehydration of 1,000 ml/day started; therefore, dehydration may have affected thrombus formation.

During surgery for COVID-19 patients, we implemented infection control measures as per the recommendations issued by the Japanese Surgical Association.^10)^ To protect medical staff from infection, we wore personal protective equipment (PPE), used N95 masks, and worked with anesthesiologists to avoid risks during tracheal intubation.

To minimize the access of medical personnel to the operating room, as many drugs and devices as possible were prepared in advance in the operating room. The postoperative transport of patients infected or suspected of being infected with the novel coronavirus was performed by a minimum number of personnel waiting outside the operating room.

Frequent handwashing and social distancing were ensured, and postoperative disinfection of operating room was performed by the waiting staff.

Because there is still little clear evidence for protection against COVID-19 infection, we consider that we should take the maximum precautions and wear the best possible equipment. In this study, there was no breathlessness during surgery owing to the use of PPE; moreover, we were able to communicate well with the staff. Fortunately, no secondary infection was observed in the hospital.

As the COVID-19 pandemic continues worldwide, additional research is required to develop preventive and treatment measures for COVID-19-related thrombosis.

## Conclusion

We report a case involving a COVID-19 patient with ALI and severe pulmonary involvement. It is important to acknowledge our experience because the COVID-19 pandemic continues worldwide. Note that ALI is treated in COVID-19 patients may be extremely difficult, and the prognosis may worsen than expected owing to their hypercoagulable state and severe respiratory failure. Additional studies are required to improve the treatment and results of ALI in COVID-19 patients.

## Abbreviations

ALI: acute limb ischemia
ARDS: acute respiratory distress syndrome
CK: creatine kinase
COVID-19: coronavirus disease 2019
CRP: C-reactive protein
CT: computed tomography
PPE: personal protective equipment
UFH: unfractionated heparin
VTE: venous thromboembolism
